# Experimental evaluation of an environmentally friendly drilling fluid for clay stabilization in shale formations

**DOI:** 10.1038/s41598-025-07888-5

**Published:** 2025-08-17

**Authors:** Ali Momeni, Seyyed Alireza Tabatabaei-Nezhad, Seyyed Shahab Tabatabaee Moradi

**Affiliations:** 1 Faculty of Petroleum and Natural Gas Engineering, Sahand University of Technology, Tabriz, Iran; 2 Faculty of Petroleum and Natural Gas Engineering, Sahand Oil and Gas Research Institute, Sahand University of Technology, Tabriz, Iran

**Keywords:** Drilling fluid, Environmentally friendly, Shale formation, Clay swelling, Glycerin, Chemical engineering, Energy, Environmental chemistry

## Abstract

To face the problems of shale drilling, oil-based fluids, mineral salts, and polymers are used, but they have limitations such as high preparation cost, hazardous environmental impact, and limited applicability in high-pressure, high-temperature environments. Therefore, introducing an environmentally friendly drilling fluid capable of inhibiting clay swelling can be beneficial for drilling shale layers and other layers containing swelling clays. Glycerin is ecologically friendly and has high stability under various environmental conditions. In the present research, a thorough set of experimental tests, including a bentonite sedimentation test, free swelling test, bentonite inhibition test, cuttings dispersion test, and visual observation of swelling, have been conducted. In all experiments, bentonite and shale cuttings were utilized as representative clay samples, with their mineralogy precisely characterized using advanced techniques such as zeta potential analysis, X-ray diffraction, and particle size distribution analysis. The representative clay samples were then brought into contact with different compositions of drilling fluids, including glycerin-based fluids and an index showing the inhibition characteristics of the fluids was reported for each test. The results indicate that glycerin-based fluids at concentrations of 100%, 90%, and 80% exhibit a significantly high capability to control clay swelling, outperforming conventional KCl-based fluids in all tests. While the 70% glycerin-based fluid showed superior performance in most tests, its results in bentonite sedimentation and cutting recovery were comparable to those of KCl-based fluids. Additionally, cutting recovery tests revealed that glycerin-based fluids not only outperform KCl-based fluids but also maintain their effectiveness more consistently with increasing temperature, highlighting their potential as a reliable and temperature-stable alternative for inhibiting clay swelling in drilling operations.

## Introduction

Shale formations account for approximately 75% of the total drilled interval in oil and gas wells worldwide^1–4^. Industrial practice shows that drilling through these formations is a challenging task in terms of borehole instability^5–8^. It should be noted that based on several studies, both reservoir transformation and drilling have the problem of reservoir wellbore instability^9,10^. On the other hand, challenging oil and gas reservoirs, such as shale oil and gas, have gradually become primary targets for exploration and development to meet the growing energy demand^11–13^. However, borehole instability in shale formations could result in a significant challenge^14^. The origin of these challenges can be primarily attributed to the significant amount of hydratable clay minerals within these formations. Hydration, swelling, and dispersion of shales during drilling operations can lead to increased costs and time of the operation^15–17^. To minimize the problems, various methods have been proposed to weaken or eliminate the interaction between clay minerals and water as the base of drilling fluid, depending on the conditions^18,19^. Drilling fluid properties play an important role in drilling operation successes^20,21^.

The inherent tendency of clay minerals to hydrate and swell can lead to various issues, including bit balling, cutting disintegration, borehole washout, pipe sticking, wellbore collapse, and inadequate hole cleaning^22–26^. As a primary solution, oil-based drilling fluids (OBDF) have been widely utilized in the industry, where an acceptable inhibition performance can be achieved by effectively removing water, therefore eliminating its contact with clay particles^27,28^. However, the application of OBDF faces limitations due to stringent environmental regulations and high initial preparation costs^29–31^. Consequently, significant research efforts have been directed toward developing environmentally friendly water-based drilling fluids to mitigate clay swelling^32,33^.

It has been proven that adding shale inhibitors to water-based fluids is an effective strategy to address shale-related challenges^34^. Mineral salts such as, potassium chloride (KCl), calcium chloride (CaCl_2_), quaternary ammonium salts, chemically modified compounds based on ammonium chloride (NH_4_Cl), glycols, silicates, nanoparticles^35^, etc., have been recommended in several studies^36–39^. However, each of these inhibitors has advantages, disadvantages, and limitations^40^. For example, potassium chloride prevents clay swelling through ion exchange mechanisms. However, high concentrations of these salts can negatively affect the environment and logging operations^41^.

Glycerol (1.2.3-propanetriol) is a sweet-tasting, odorless, colorless liquid with a high viscosity at room temperature. Under typical environmental circumstances, glycerol has excellent stability and is compatible with a wide range of other additives. Given the special properties of glycerol, it can be a possible replacement for conventional drilling fluids^42–46^.

There are several studies in the literature that have been undertaken to investigate the efficacy of glycerin as a base fluid or as an additive within drilling fluid formulations. In a study conducted by Cheatham et al.^47^, the transportation of cuttings within fluids composed of water and glycerin was investigated to analyze the influence of viscosity on cutting transport performance. Similarly, Philip et al.^48^ investigated the proportion of uplifted drilling cuttings in base fluids containing varying volume percentages of water and glycerin. Their findings demonstrated more efficient cutting transport at higher glycerin concentrations. In some other studies, glycerin has been employed not as an additive to drilling mud but as the primary base fluid to evaluate its effectiveness in facilitating cutting transport^49–55^.

Marbun et al.^46^ investigated the impact of glycerin addition on the properties of drilling fluids. Their findings revealed that the addition of glycerin improved the drilling fluid characteristics, such as lubricity and filter cake thickness. Other studies proved the effect of glycerin additive on the enhancements of drilling fluid filtration and rheological properties^43,45^. Plastic viscosity, yield point, and gel strength are the most important rheological parameters of drilling fluid^56,57^.

Duarte et al.^58^ addressed the dissolution of hydrocarbon gases in oil-based drilling fluids and the associated challenges, such as delayed kick detection and subsequent well control issues. They also studied the solubility of methane in glycerin-based fluids as an alternative. The experimental results indicated that, unlike OBDF, glycerin-based fluids exhibit complete environmental compatibility and demonstrate superior performance in mitigating gas solubility-related challenges.

As can be seen, the effect of glycerin addition on the inhibition characteristics of drilling fluids is not fully covered in previous studies. To address this gap, this study focuses on evaluating the inhibition performance of glycerin for reducing shale swelling. Therefore, a comprehensive suite of experimental tests, including bentonite sedimentation test, bentonite inhibition test, particle size distribution analysis, cuttings dispersion test, and free swelling test, was designed and conducted. Also, advanced characterization techniques, including zeta potential analysis, X-ray diffraction (XRD), and particle size distribution analysis, were employed to examine the properties of the representative shale samples.

## Materials and methods

### Materials

**Fig. 1:**
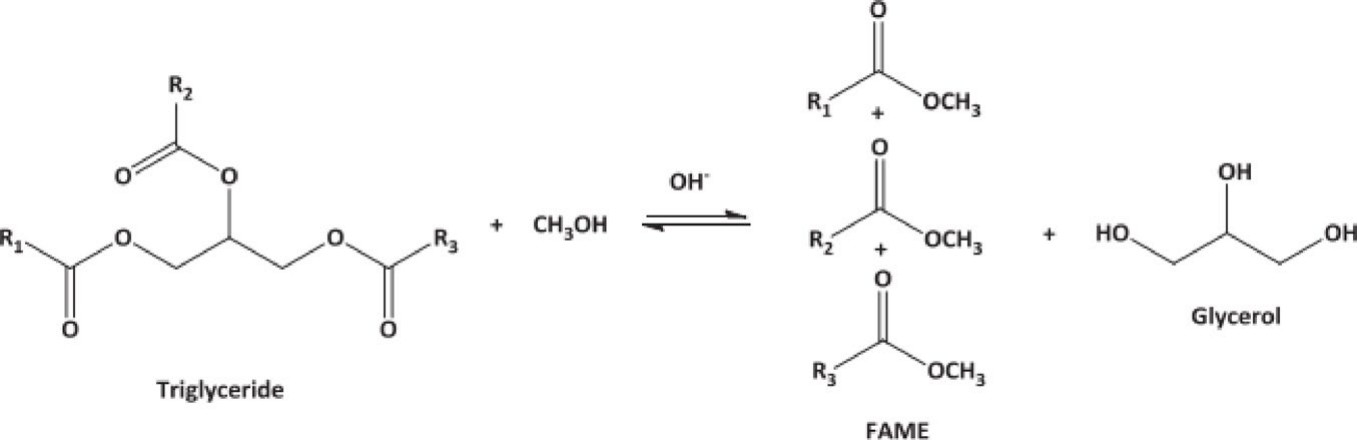
Production of glycerol from petrochemical process^59^.

For the preparation of base fluids, deionized water and synthetic glycerin (Fig. 1), with a purity of 99.5% were used. Table 1 provides a summary of glycerin’s main characteristics. Glycerin is almost non-irritating, and there is no known negative impact of glycerin on the environment. Additionally, three hydrophilic alcoholic hydroxyl groups in the glycerol chemical structure contribute to its hygroscopic character and water solubility. The compositions of the base fluids utilized for tests are presented in Table 2.

**Table 1:** Physicochemical glycerol properties in room temperature conditions (20 °C)^60^.

Chemical formula	C3H5(OH)3
Density	1.261 g/cm^3^
Molecular mass	92.09382 g/mol
Surface tension	64.00 mN/m
Viscosity	1.5 Pa s
Flashpoint	160 C
Melting point	18.2 C
Boiling point	290 C
Food energy	4.32 kcal/g

**Table 2:** Composition of base fluids.

	A*	*B*	*C*	*D*	*E*	*F*
Glycerin (mL)	0	0	280	320	360	400
Deionized water (mL)	400	400	120	80	40	0
Glycerin:Water ratio	0:100	0:100	70:30	80:20	90:10	100:0

A* base fluid added with 5% wt. KCl.

Bentonite and drilling cutting samples from the Pabdeh formation were used as the representative shale samples in this study. Bentonite, composed of montmorillonite clay mineral, is known for its significant swelling and dispersion properties. The Paleocene to Oligocene Pabdeh Formation is mainly composed of gray/brown shales and marls with calcite and dolomite interlayers, serving as both a prominent source rock and an effective cap rock within the Dezful Embayment^61,62^. The high content of reactive clay minerals in the formation renders it particularly susceptible to swelling upon interaction with water-based fluids. This phenomenon often leads to operational challenges, including wellbore instability, reduced drilling efficiency, and increased non-productive time during drilling operations^63,64^. Consequently, the oil and gas industry is particularly concerned about the Pabdeh Shale Formation, which necessitates the development of specialized drilling fluid systems and mitigation strategies to address clay swelling and ensure optimal wellbore stability in this geologically complex region.

### Experiments

**Fig. 2:**
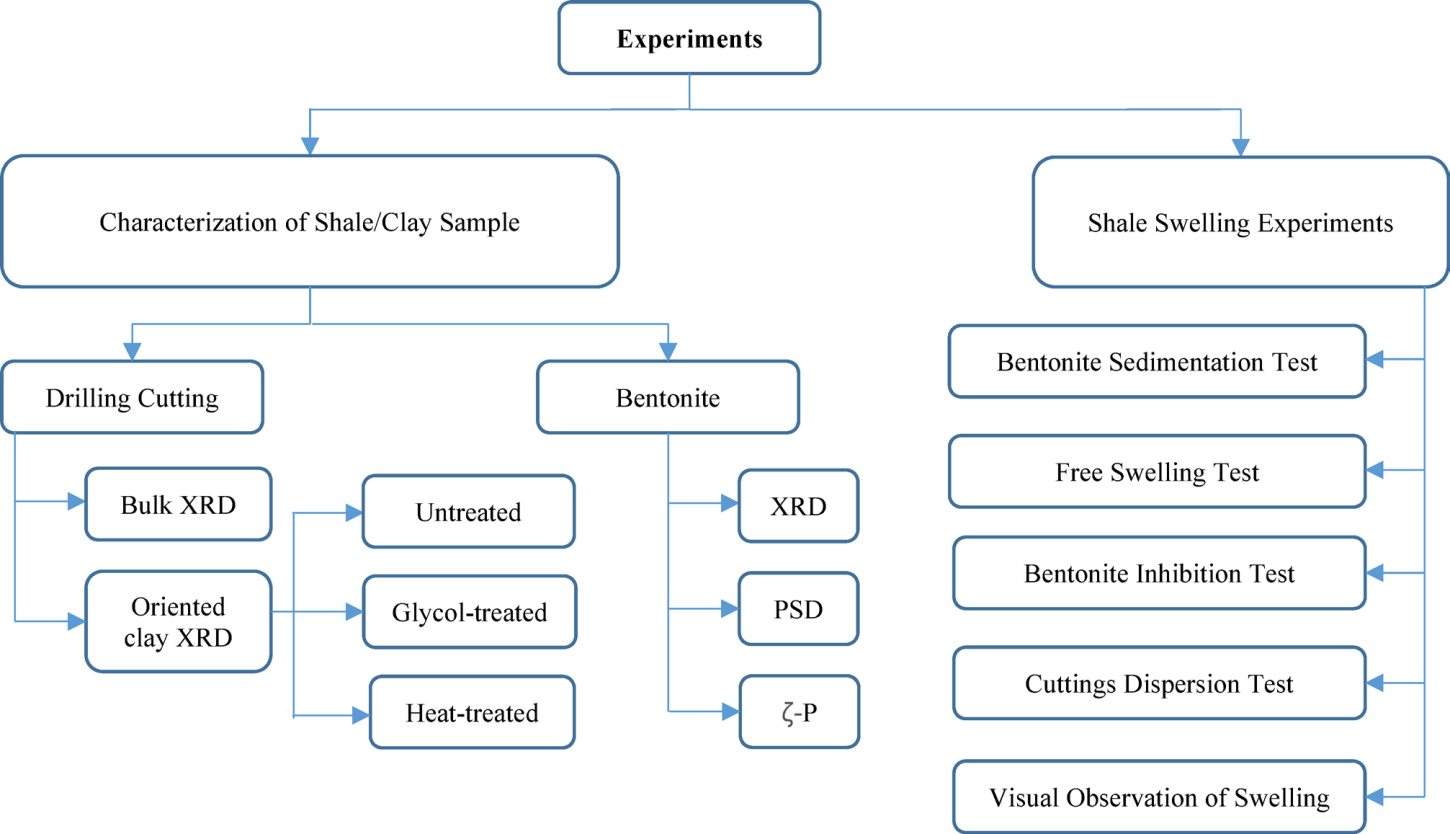
Schematic of experiments conducted in this study.

Several laboratory methods have been developed to characterize clay properties and assess their reactivity and sensitivity to drilling fluids. These tests are often performed on bentonite, drilling cuttings, or shale cores^65^. The overview of experimental tests conducted in this study is presented in Fig. 2.

### X-ray diffraction (XRD)

The X-ray diffraction (XRD) technique, frequently employed to determine the types of minerals in a sample, involves rotating a sample and simultaneously bombarding it with X-rays. The mineral’s crystalline structure scatters X-rays with similar characteristics that are detected by the device. The peaks obtained at 2θ can help identify the elements present in the composition^66^. This test was conducted on bentonite and drilling cuttings of shale formation.

### Bentonite ζ- potential and particle size distribution

Ζ-potential, as a type of electrical potential, is measured at the solid/liquid interface of colloidal systems^67,68^ and can be used to measure the relative surface charge. To prepare the samples for this test, 0.2 g of bentonite sample was added to 100 ml of distilled water and mixed at 1000 rpm for 20 min to create a homogeneous system. Then, the size of clay particles suspended in the fluid can be measured. This test was performed using the phase analysis light scattering (PALS) technique to measure ζ-potential and particle size distribution with a Malvern zetasizer device.

### Characterization of drilling cuttings

Drilling cuttings from the Pabdeh formation were characterized through a systematic and detailed experimental procedure to identify and analyze the mineralogical composition, with particular focus on the clay minerals present in the sample. For this purpose, drilling cuttings were collected from the wellbore, carefully washed to remove any residual drilling fluid or contaminants, and subsequently dried in an oven at a constant temperature of 60 °C until complete moisture removal was achieved. Following the drying process, 20 g of the cuttings were finely ground into a homogeneous powder using an agate mortar to minimize contamination and ensure sample consistency. The powdered samples were then subjected to XRD analysis to identify the clay minerals present in the bulk shale sample.

To investigate the clay mineralogy in greater detail, the oriented clay sample preparation method was employed. This method, recommended by Moore and Reynolds^69^ is used to separate clay-sized particles (particles smaller than 2 microns) from the bulk sample^70^. The separation was carried out suing sedimentation techniques, which exploit the differential settling rates of particles based on their size. Once the clay-sized fraction was isolated, oriented clay samples were prepared for XRD analysis under three distinct conditions: untreated, glycol-treated (at 60 °C), and heat-treated (at 550 °C).

This comprehensive approach provides a thorough understanding of the clay mineralogy of the drilling cuttings, leading to critical insights into their behavior and reactivity under different conditions.

### Bentonite sedimentation test

When bentonite is exposed to water, its platelets separate and form colloids in water^71^. If the fluid surrounding the clay minerals has inhibiting properties, the formed colloids will be unstable, causing the clay platelets to bind together and settle. A bentonite sedimentation test can be used to measure the instability of clay minerals in inhibitive environments^72,73^. To conduct this test, after preparing 250 cc of the different drilling fluids, 5% by weight of bentonite was added to each fluid, and the mixture was stirred at 2000 rpm for 20 min to form a homogeneous suspension. Then, 200 cc of the resulting suspensions were poured into graduated cylinders. To mitigate water evaporation and prevent subsequent alterations in the composition ratio, 5 cc of paraffin was added to the each mixture. After enough time (at least 48 h), a distinct contact surface is formed between the settled bentonite and the overlying mixture. The lower the contact surface, the better the inhibition performance of the solution. The experiments were performed at two distinct temperatures, 25 °C and 75 °C, to investigate the influence of temperature on the observed outcomes.

### Free swelling test

The purpose of this test is to measure the free swelling of a shale sample after being exposed to drilling fluid. In this method, a shale sample (e.g., powdered cuttings from a shale formation) or bentonite as a swellable clay mineral is placed into graduated cylinders in equal amounts^74,75^. In this study, 30 g of bentonite were added into 200 ml graduated cylinders and brought in contact with base fluids. The amount of swelling is analyzed by measuring the increase in volume of the clay sample. The results are presented as a graph showing the percentage of swelling versus time. A high final swelling percentage indicates the drilling fluid’s inability to prevent swelling. This test was also performed at two distinct temperatures, 25 °C and 75 °C, to investigate the influence of temperature on the observed outcomes.

### Bentonite inhibition test

The bentonite inhibition test is typically employed as a screening technique to evaluate the ability of a drilling fluid formulation to prevent bentonite swelling and maintain poor rheological properties. This test determines the maximum amount of API bentonite that can be inhibited by a shale inhibitor. In this test, 20 g of bentonite was added daily to 400 mL of base fluid, and after being subjected to hot-rolling conditions at 75 °C for 16 h, the rheological properties were measured. This process was repeated until the system becomes too thick for rheology measurements^76,77^.

### Cuttings dispersion test

This test measures the tendency of the shale sample to disperse after exposure to the drilling fluid^78,79^. In this test, hot rolling was used to investigate the effect of the drilling fluid on the shale sample. For this purpose, 20 g of dried cuttings with particle sizes between 2 and 4 mm, along with the drilling fluid, were placed in a hot-rolling cell. After hot rolling the cell for 16 h at 75 °C, the shale cuttings were recovered, washed, and dried in an oven at 105 °C for 24 h. Then, to determine the percentage of cutting recovery, the sample was weighed after screening with a 0.5 mm pore-size sieve. The results are presented as the percentage of cutting recovery. Low values of cutting recovery indicate poor inhibition performance of the drilling fluid against dispersion, as a larger amount of cuttings is dispersed in the drilling fluid. This process was repeated at 45 °C and 60 °C to study the effect of temperature on cutting recovery.

### Visual observation of swelling

This experiment was conducted to qualitatively assess the swelling behavior of a clay sample (bentonite) when exposed to different base fluids^80,81^. To achieve this, clay tablets were fabricated using a hydraulic press machine under a pressure of 15,000 psi, with each tablet standardized to a diameter of 1 cm and a thickness of 2 mm. These tablets were subsequently immersed in the respective base fluids, and their swelling characteristics were monitored and documented over a time. The visual observations provided critical insights into the inhibitory effects of the base fluids on clay swelling, contributing to a comprehensive understanding of their performance in mitigating hydration and expansion of bentonite.

## Results and discussion

In this section, the results of the laboratory work are presented and discussed.

### X-ray diffraction (XRD) of bentonite

**Fig. 3:**
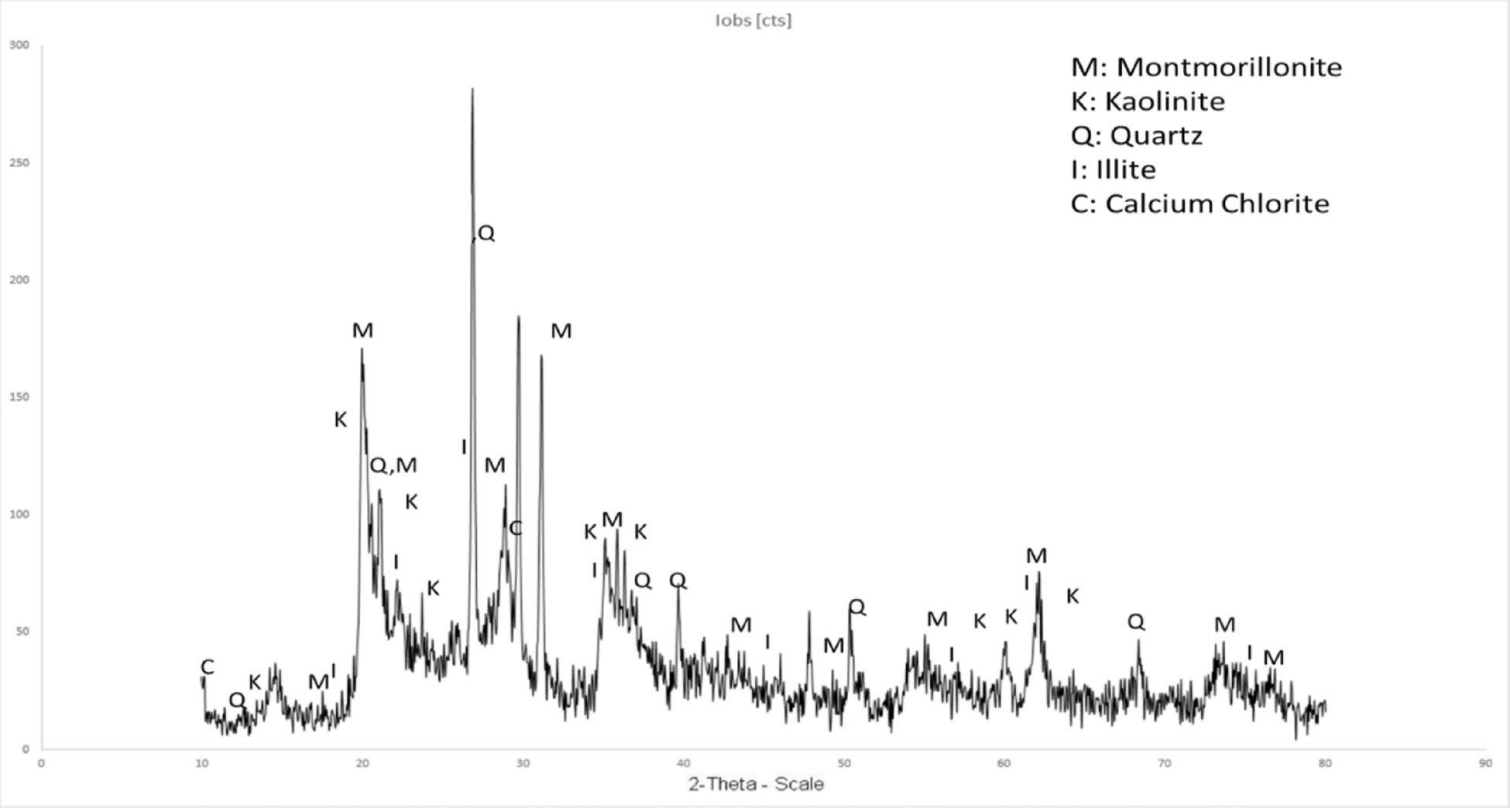
XRD results of bentonite used in this study, which mainly consist of montmorillonite, kaolinite, illite, and quartz.

The XRD analysis of the bentonite sample revealed a high proportion of swellable clay minerals, predominantly montmorillonite, along with kaolinite, illite, and quartz (Fig. 3). XRD results also indicated the presence of other minerals, such as quartz and calcium chlorite, which are commonly found in bentonite. Consequently, it can be inferred that the bentonite used in the various tests is an ideal sample for such investigations due to the presence of highly swellable clay minerals.

### Bentonite ζ-potential and particle size distribution

**Fig. 4:**
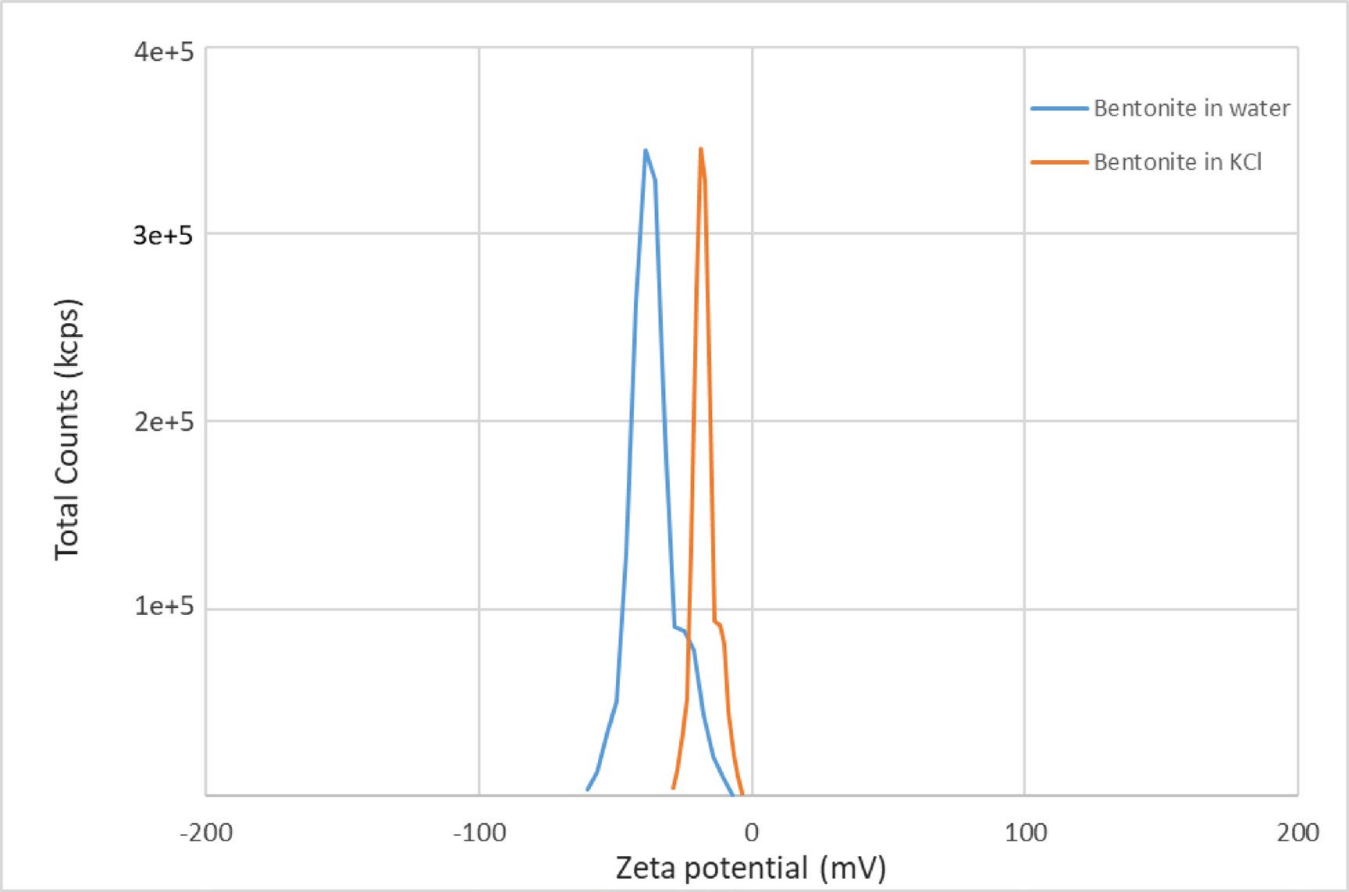
ζ-Potential distribution profile for the bentonite sample.

**Fig. 5:**
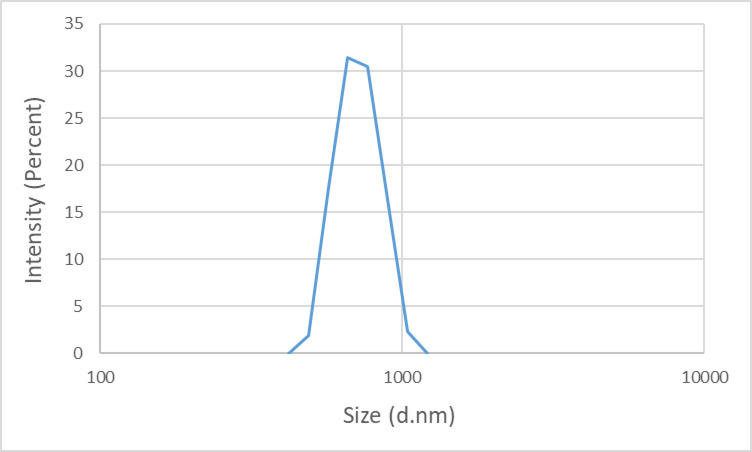
Particle size distribution analysis of the bentonite sample.

The results of the ζ-potential analysis are illustrated in Fig. 4. The observed negative ζ-potential values can be attributed to the inherent negative surface charge of the clay platelets^82^. The mean ζ -potential was measured at − 36.1 mV, while the wall ζ-potential was recorded at − 38.68 mV. These values, as shown in Fig. 4, indicate the negative surface charge of the particles, which is aligned closely with the expected ζ-potential range for this category of swelling clays, thereby confirming that the sample selected for the swelling is appropriate and representative of the material class under investigation^82,83^. Furthermore, the results of the zeta potential of bentonite in KCl-based fluid indicate that the presence of potassium chloride salts in water has led to a decrease in the negative surface charge of clay minerals until − 18.63 mV. The particle size distribution analysis of the bentonite suspension in water further demonstrates that the particles exhibit rapid interaction with water, leading to the separation of clay platelets and their subsequent breakdown into smaller particles (Fig. 5).

### Characterization of drilling cuttings

**Fig. 6:**
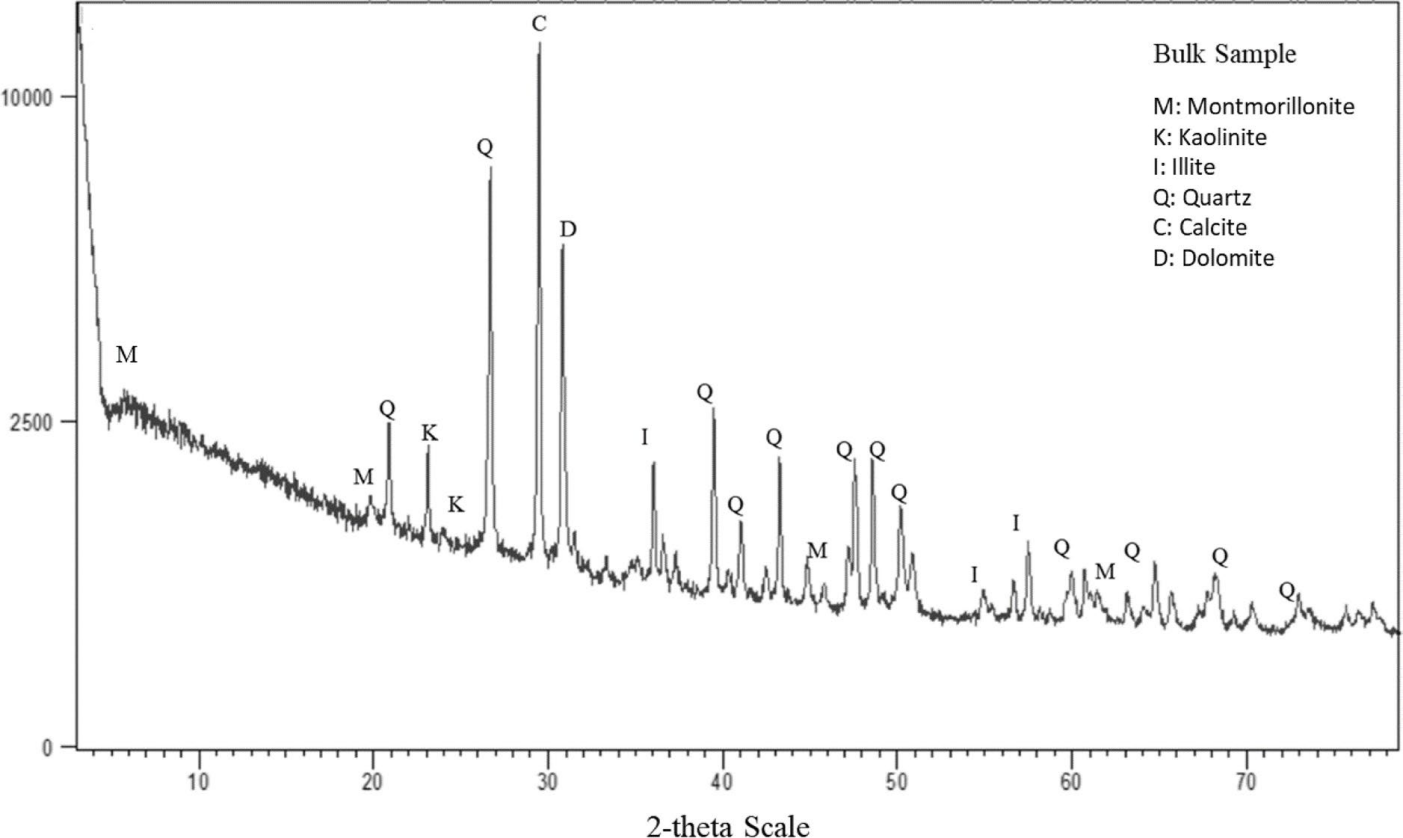
X-ray diffraction (XRD) analysis results for the bulk sample, illustrating the mineralogical composition of drilling cuttings.

The results of the XRD analysis (Fig. 6) conducted on the drilling cuttings indicate the presence of clay minerals such as montmorillonite, kaolinite, and illite in the sample. The outcomes demonstrate that the chosen sample is highly suitable for inhibition testing, as it contains a representative composition of reactive clay minerals.

As mentioned before, the Pabdeh Formation is recognized as a problematic formation in drilling operations. The presence of water-sensitive clays, such as montmorillonite, in the XRD results, provides a clear explanation for the operational challenges encountered in this formation. These issues typically arise when the drilling fluid lacks sufficient inhibitory properties to prevent clay swelling, leading to wellbore instability and reduced drilling efficiency. The findings emphasize the need to develop drilling fluids with effective inhibition capabilities to address the inherent reactivity of clay minerals in such formations.

**Fig. 7:**
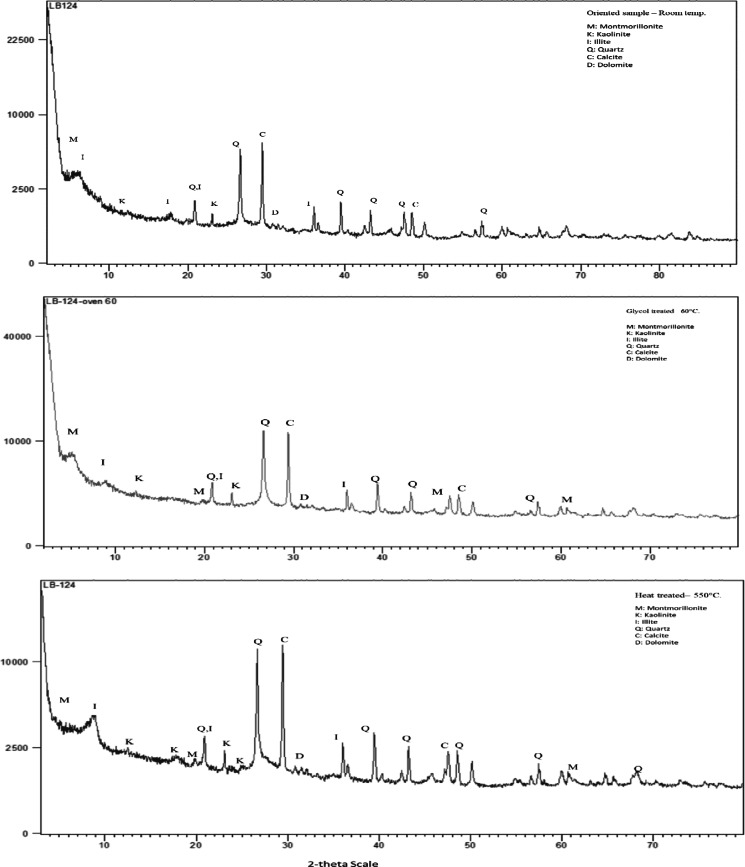
X-ray diffraction (XRD) analysis results for the oriented clay sample, presented in sequential order from top to bottom: untreated, glycol-treated, and heat-treated conditions, respectively.

Furthermore, the XRD analysis of the oriented clay sample confirmed the presence of water-reactive clay minerals (Fig. 7). Therefore, XRD analysis of both bulk and oriented clay samples has provided critical insights into the mineralogical composition of the Pabdeh Formation.

### Bentonite sedimentation test

**Fig. 8:**
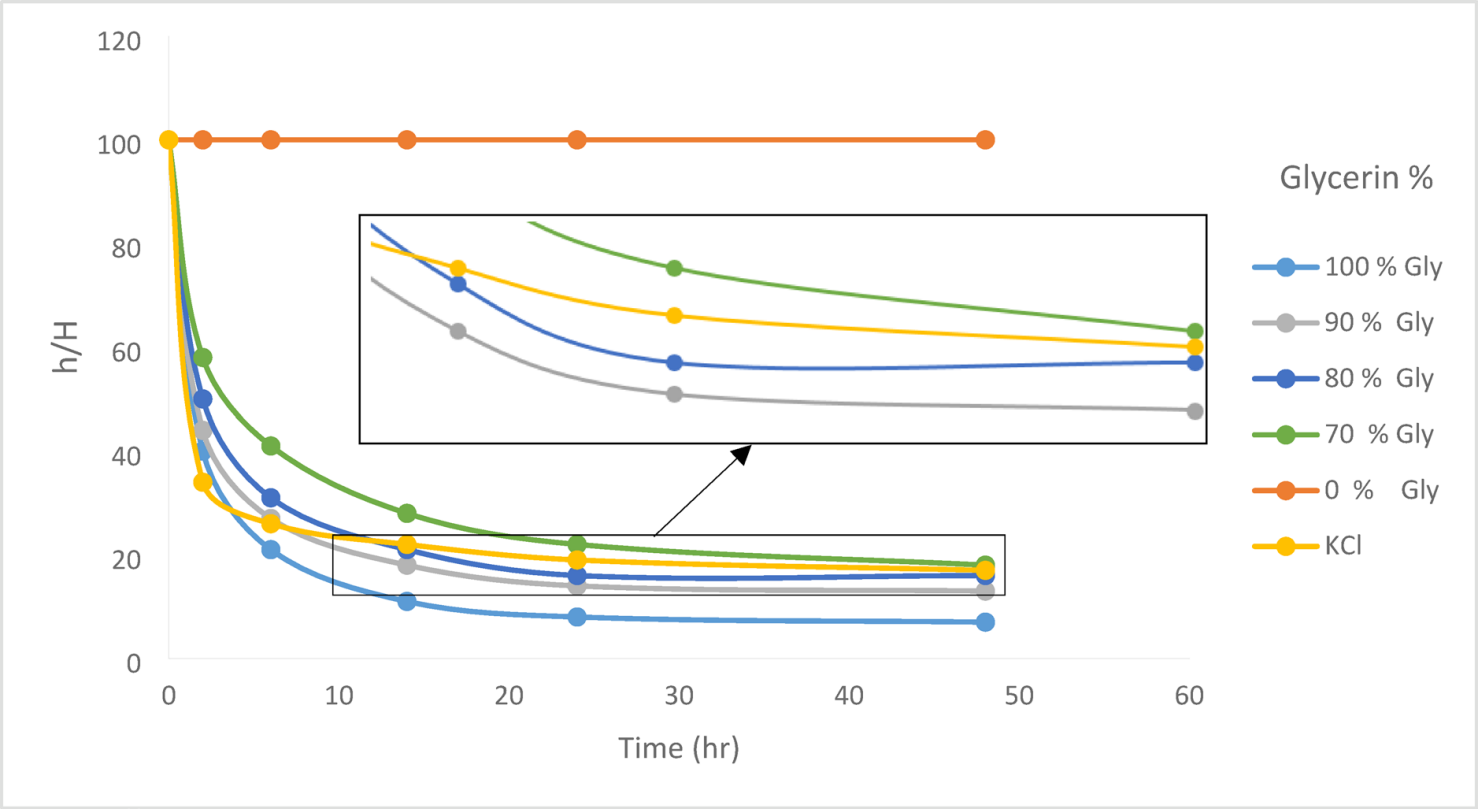
Results of Bentonite Sedimentation Test, ratio of Bentonite sediment height to the initial height versus time.

**Fig. 9:**
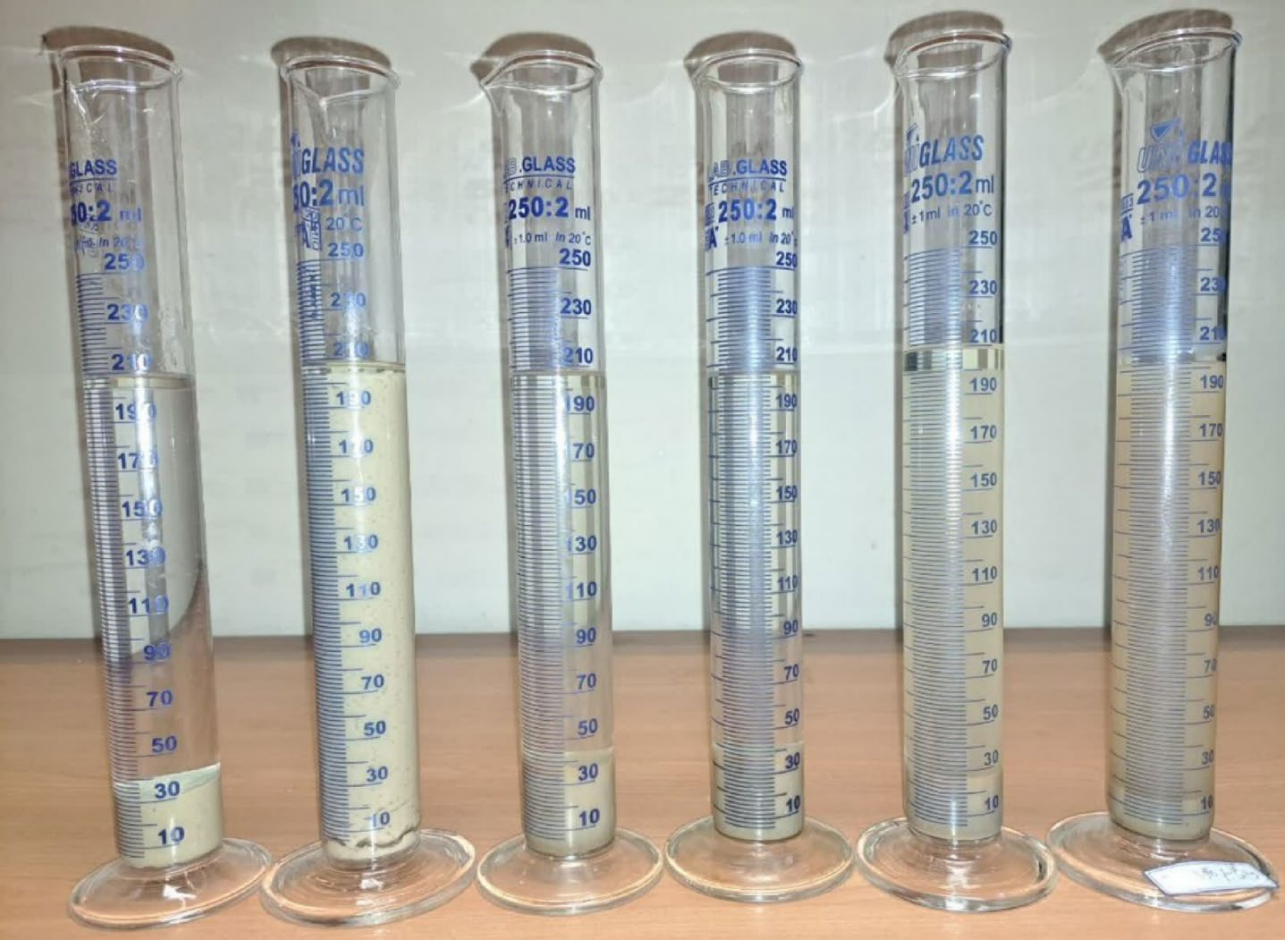
Bentonite sedimentation test after 16 h. (from left to right KCl, 0, 70, 80, 90, 100 glycerin samples).

The results of bentonite sedimentation tests at 75 °C are shown in Fig. 8. As can be seen, the glycerin based fluid has been able to prevent the separation of clay platelets from each other effectively and has shown good inhibition (Fig. 9). In this test, after 48 h, the base fluid sample without any inhibitor maintained its stability in water, and two separate phases were not formed. Therefore, its height was considered 100% at the end of the test. Sedimentation in the KCl-based fluid occurred at a faster rate, which could be attributed to the lower viscosity of the base fluid. However, the final height of the settled bentonite after 48 h was lower in the base fluids with 100%, 90%, and 80% glycerin demonstrating superior inhibition performance compared to the KCl-based fluid. However, in the 70% glycerin concentration, the height was nearly equivalent to the KCl sample test, with both demonstrating comparable inhibition performance.

### Free swelling test

**Fig. 10:**
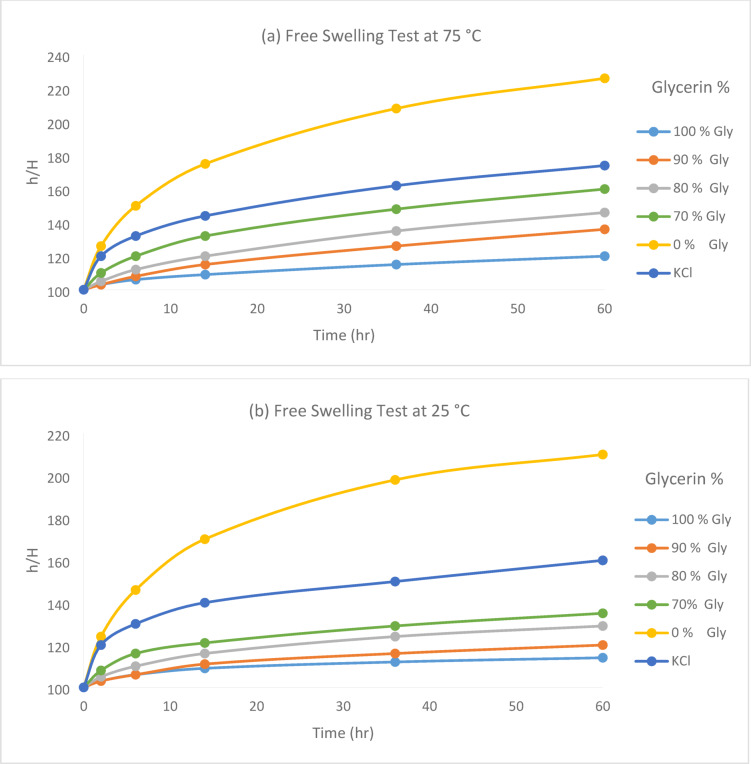
Results of free swelling test at (**a**) 75 °C and (**b**) 25 °C.

**Fig. 11:**
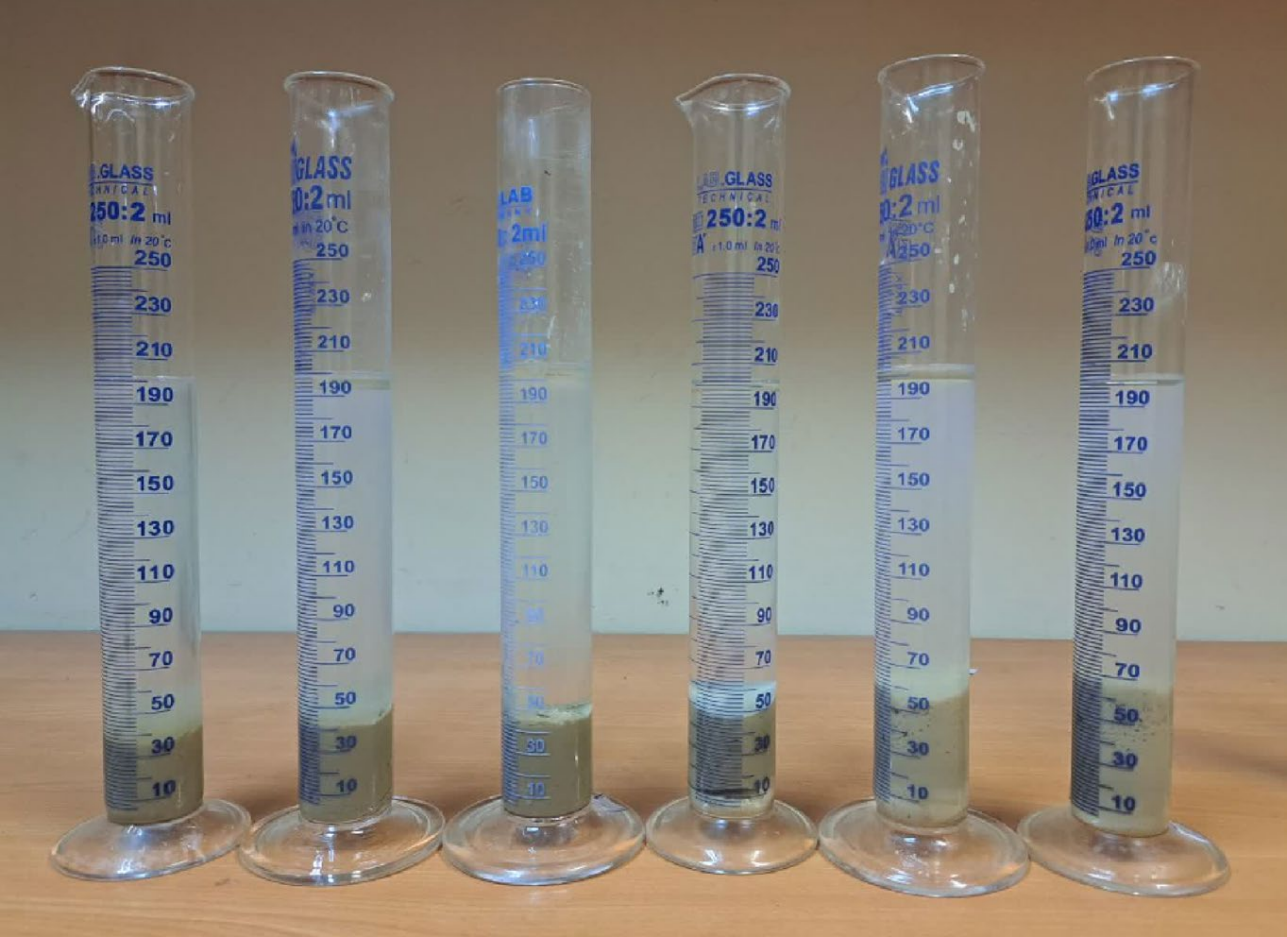
Free swelling test performed with different base fluids.

The results of this test also support the significant effect of glycerin on the inhibition of the clay minerals. Figures 10 and 11 show that the free swelling of clay particles in the base fluids with 100%, 90%, 80%, and 70% glycerin/water was minimal, and these fluids effectively inhibited clay swelling. In the base fluid without glycerin and KCl, the swelling of clay particles was significant, and the amount of free swelling in this sample increased to more than twice the initial height after 60 h. The KCl-based fluid also prevented clay swelling, although its performance was lower than that of the glycerin. With an increase in temperature, a corresponding rise in swelling was observed; however, at both 25 °C and 75 °C, the glycerin-based fluid demonstrated superior performance in controlling swelling compared to KCl-based fluids.

### Bentonite inhibition test

**Fig. 12:**
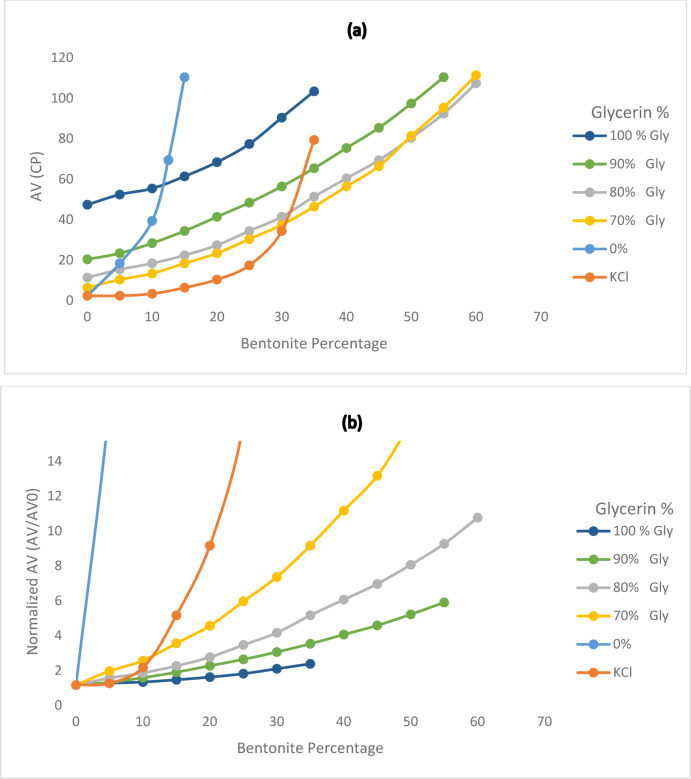
Variation of (**a**) apparent viscosity and (**b**) normalized apparent viscosity as a function of bentonite concentration in the bentonite inhibition test for various base fluids.

**Fig. 13:**
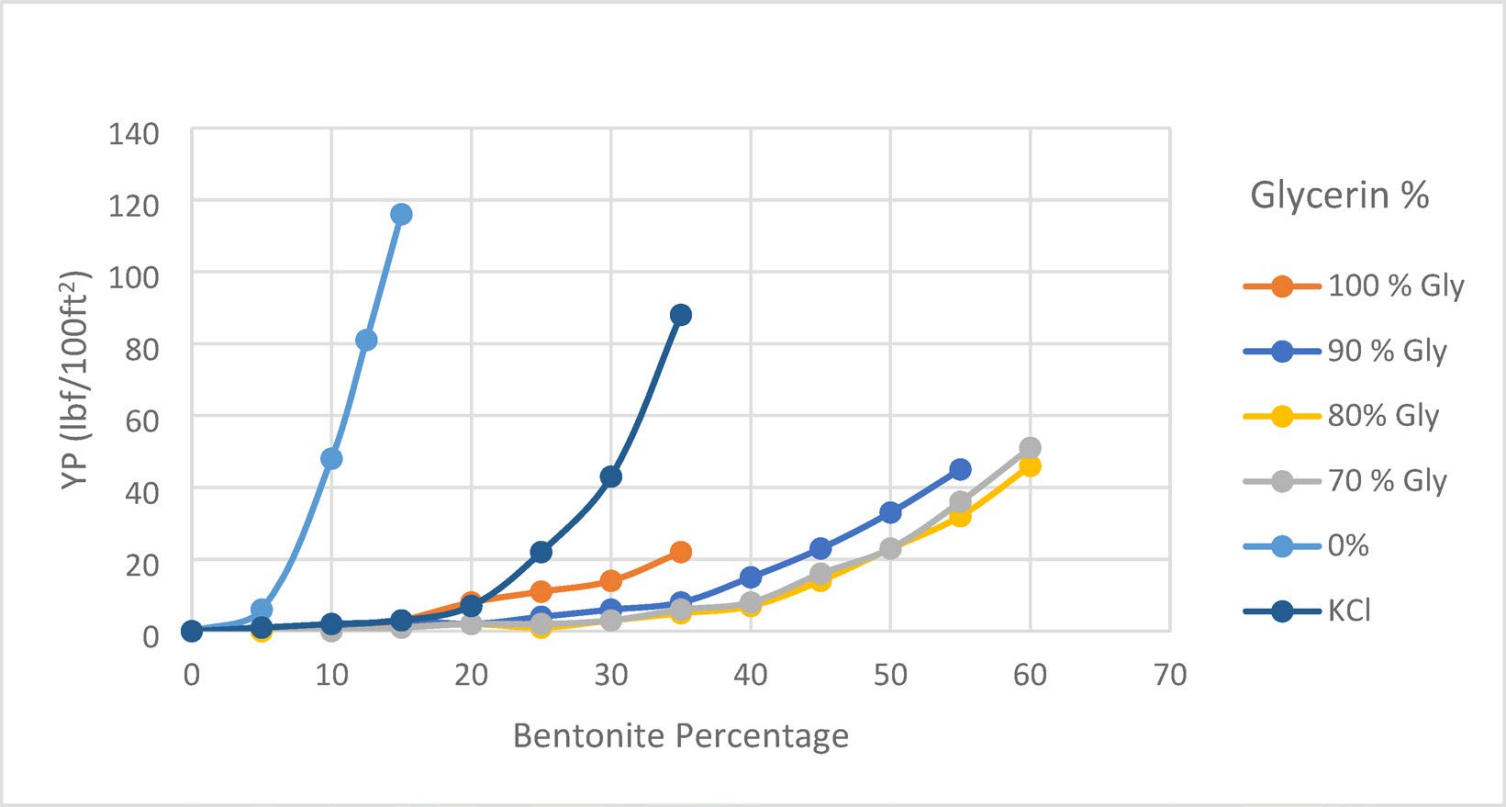
Variation of yield point as a function of bentonite concentration in the bentonite inhibition test for various base fluids.

As shown in Figs. 12 and 13, the results demonstrate the superior performance of glycerin-based fluids, especially at higher concentrations. Notably, the high viscosity of the base fluid led to fewer procedural steps in the test, as the elevated initial viscosity of the glycerin-based fluid inherently limited the amount of bentonite that could be incorporated into the system. Consequently, the lower bentonite uptake observed for the 100% glycerin-based fluid does not indicate poor clay swelling inhibition, but rather reflects the fluid’s high viscosity, restricting bentonite integration. Figure 12b illustrates the normalized apparent viscosity, which verifies that glycerin-based fluids at higher concentrations have better inhibition performance.

When the influence of initial viscosity is disregarded, the highest bentonite obtained values were recorded for the 80% and 70% glycerin-based samples. This suggests that these concentrations may represent an optimal balance between viscosity and inhibition efficiency. Notably, across all tested samples, glycerin-based fluids at concentrations of 100%, 90%, 80%, and 70% consistently demonstrated superior bentonite inhibition performance compared to the KCl-based fluid.

### Visual observation of swelling

The results of this experiment are presented in Table 3. As illustrated, the interaction between the 0% base fluid (without glycerin) and the clay tablet commenced rapidly at the onset of the experiment. This interaction, though less pronounced, was also observed in the 70% glycerin and KCl samples, ultimately leading to the deformation of the clay tablet. In contrast, no significant interaction was detected for the 90% glycerin-based sample during the initial 20 min or for the 100% glycerin-based sample within the first 2 h. For the 80% glycerin-based sample, the interaction between the fluid and the clay tablet began approximately 10 min after the experiment started; however, the rate of this interaction was significantly slower compared to the 70% glycerin and KCl samples.

**Table 3:**
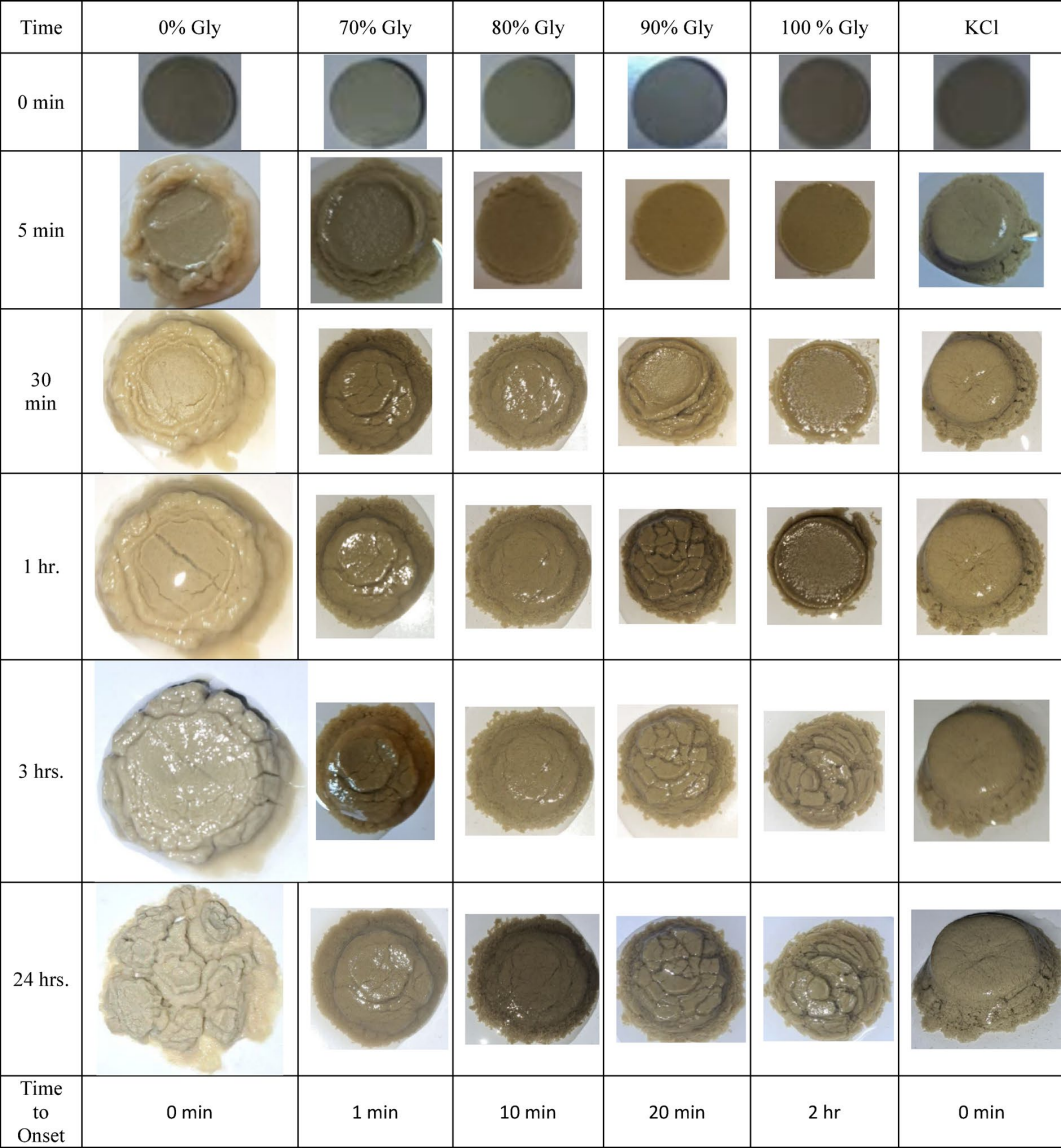
Visual observation of swelling rate in 24 h.

A critical observation is the exceptional performance of the 100%, 90%, and, to a lesser extent, 80% glycerin-based samples. By the conclusion of the experiment, the swelling of the clay tablet in these samples was minimal, with time primarily resulting in fluid penetration and slight deformation of the tablet. In general, glycerin-based fluids demonstrated highly effective performance in inhibiting the swelling of clay minerals.

### Cuttings dispersion test

**Fig. 14:**
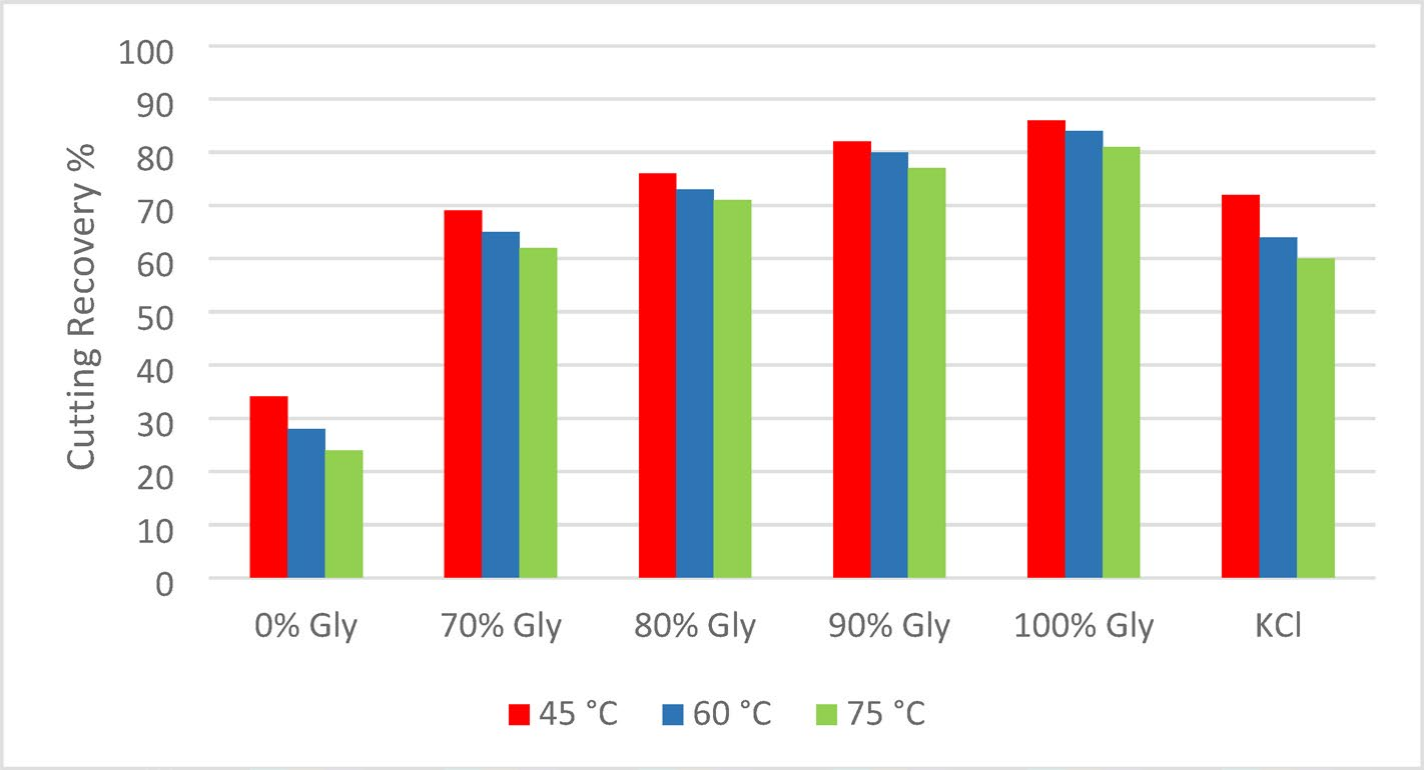
Results of cuttings dispersion test in different temperatures.

As shown in Fig. 14, the experimental findings from this test reveal that base fluids formulated with 100%, 90%, and 80% glycerin concentrations demonstrated superior performance in terms of drilling cutting recovery efficiency. It’s worth noting that the 100% glycerin-based fluid consistently achieved cutting recovery rates exceeding 80% across the entire range of tested temperatures, which underscored its robust performance under varying thermal conditions. In comparison, the 70% glycerin-based fluid displayed performance metrics that were closely aligned with those of the KCl-based fluid.

A consistent trend observed across all experiments was a reduction in cutting recovery as temperature increased. However, the detrimental effect of elevated temperature on inhibition efficiency was significantly less in glycerin-based fluids compared to their KCl-based counterparts. This differential response highlights the enhanced thermal stability of glycerin-based fluids, which enables them to maintain effective inhibition performance even under high temperature conditions.

In this study, the interaction between shale/clay minerals and drilling fluid was investigated through a comprehensive series of precise laboratory experiments. The results demonstrated the exceptional performance of the suggested fluid in controlling clay swelling. All experimental measurements were conducted with strict precision (± 0.01 g, ± 1 mL, ± 1 °C), and statistical significance was verified via one-way ANOVA (α = 0.05) for triplicate trials. It is widely acknowledged that the interaction between drilling fluids and shale formations containing reactive clays can be inhibited or minimized through one of three main mechanisms: ionic inhibition, physical plugging, and encapsulation^5,84^. In the ionic inhibition approach, the focus is on interlayer cations, which contribute to the chemical stability of clay minerals. Consequently, when salt is utilized, the objective is to reduce cation exchange by increasing the salt concentration within the drilling fluid^85–87^. In oil-based fluids, ion exchange is inherently minimized due to their OBFs nature^88^. The physical plugging method involves the use of nanoparticles to block the pores of clay minerals, thereby preventing fluid penetration^89–91^. In the encapsulation approach, a protective layer is formed on the surface of clay minerals, effectively shielding them from fluid interaction^92,93^. Given that the first two mechanisms are inconsistent with the properties of glycerin, the inhibition of clay swelling in glycerin-based fluids is probably achieved through the third mechanism. Glycerin also has can form hydrogen bonds with water^94^. This phenomenon may also explain the inhibition of drilling fluids containing glycerin through encapsulation. However, to achieve a more in-depth and comprehensive understanding of the dominant mechanisms responsible for inhibiting the swelling of clay minerals, additional research is essential. Future studies should focus on systematically exploring the physicochemical interactions between clay minerals and inhibitory agents, as well as evaluating the influence of varying environmental conditions.

## Conclusion

Drilling shale formations is often challenging due to related problems. The use of oil-based fluids, mineral salts, and polymers also faces problems such as high cost, environmental problems, and temperature and pressure limitations. Glycerin, on the one hand, is environmentally friendly and, on the other hand, has high stability under various environmental conditions, including HPHT conditions. In this study, through various tests, such as bentonite sedimentation test, free swelling test, bentonite inhibition test, cuttings dispersion test, and visual observation of swelling, it was shown that glycerin-based drilling fluids exhibit a significant capacity to inhibit clay swelling. A comprehensive suite of advanced techniques was employed to characterize representative clay samples, including bentonite and shale cuttings. Characterization tests, such as zeta potential analysis, X-ray diffraction (XRD) analysis, and particle size distribution analysis for bentonite, as well as XRD of bulk and oriented clay samples in three distinct conditions—untreated, glycol-treated, and heat-treated—confirmed that the selected clay samples were not only ideal for robust laboratory inhibition testing but also highly applicable to real-world drilling operations, offering practical solutions to mitigate clay swelling problems.

The results indicate that glycerin-based fluids at concentrations of 100%, 90%, and 80% exhibit a significant ability to control clay swelling, consistently outperforming conventional KCl-based fluids across all tests. Although the 70% glycerin-based fluid demonstrated superior performance in most tests, its results in bentonite sedimentation and cutting recovery were comparable to those of the KCl-based fluid. Furthermore, cutting recovery tests revealed that glycerin-based fluids not only surpass the performance of KCl-based fluids but also retain their effectiveness with increasing temperature. The visual observation of the swelling test provided additional objective confirmation of the exceptional performance of glycerin-based fluids in swelling inhibition. These findings underscore the potential of glycerin-based fluids as a reliable and temperature-stable alternative for effectively controlling clay swelling in drilling operations.

For further studies, it is suggested to investigate the physicochemical interaction between clay minerals and inhibitory agents, as well as various environmental conditions, including HPHT, which were not included in this study due to the large volume of experiments, the breadth of the subject, and laboratory limitations.

## Data Availability

The datasets generated and/or analysed during the current study are available in the Cambridge Crystallographic Data (CSD) repository, under deposition number 2455468.
